# The Acute Needs for Palliative Care Services in Ethiopia

**DOI:** 10.4314/ejhs.v33i6.1

**Published:** 2023-11

**Authors:** Endalew Hailu, Tekle Ferede, Neguss Yilma

**Affiliations:** 1 School of Nursing, Institute of Health, Jimma University, Ethiopia; 2 Palliative Care Focal Person at Jimma University Medical Center, Jimma University, Ethiopia; 3 Department of English Language literature and Literature, College of Social Sciences and Humanities, Jimma University, Ethiopia

Palliative care, an approach used to improve the quality of life of patients with life-limiting illnesses and their families through identification, assessment and treatment of pains (physical, emotional, psychological or spiritual), is an important part of the World Health Organization's agenda (1). WHO's target for Sustainable Development Goal 3 (2016-2030) is to devise and implement palliative care policies for 40 million people. However, only 14% of the global population was reported to be receiving palliative care (2). More strikingly, palliative care, which is claimed to be a basic human right (3), is inaccessible to the majority of the population in LIMCs, including Ethiopia, which are characterized with astounding numbers of patients seeking the care (4).

Accordingly, emerging circumstances and research findings show that there are extensive unmet palliative care needs in Ethiopia. A study (5) revealed that 95.5% of oncology patients in Ethiopia were diagnosed with moderate or severe pain, and of these, 24% were not prescribed analgesia. According to this study, more importantly, 80% of the non-communicable diseases were reported as severe pain. To make matters worse, a considerable number of severe cases, including cancer and HIV/AIDS, can be left untreated at all. Untreated pain and high costs of illness have been found to be major contributors to psychosocial distress, exacerbating the conditions of patients and exposing them to poor quality of life (5). These are among the instances that vividly show the acute need for palliative care services in Ethiopia.

Furthermore, for decades, Ethiopia has been suffering from repeated catastrophes such as drought, famine, poverty and ongoing armed conflicts. Especially, armed internal conflicts, which are intensifying in recent years, have been worsening human plight and agony, resulting in continued humanitarian tragedies. Within this scenario, millions of Ethiopians have either little or no access to well-resourced healthcare facilities. Armed conflicts are known to be significant causes of deadly injuries and various traumas, which require healthcare interventions that go beyond the provision of curative treatments. In other words, in humanitarian crises situations, holistic healthcare approaches, the notable one being palliative care, are vital to sooth the physical, emotional and psychological sufferings of the vulnerable populations.

This being the case, however, there exist formidable challenges in palliative care delivery in humanitarian crisis circumstances. For example, a systematic review was conducted on the palliative literature (2005–2017 focusing on humanitarian situations (e.g., disasters, armed conflicts, epidemics). The review was done to describe palliative care needs, practices, barriers and recommendations during humanitarian crises. The review concluded that 95% per-reviewed and gray literatures revealed a scarcity of data on palliative care needs and interventions provided in crises, challenges of care provision particularly due to inadequate pain relief resources and guidelines, a lack of consensus on the ethics of providing or limiting palliative care as part of humanitarian healthcare response, and the importance of contextually appropriate care (6).

Millions of Ethiopians in war-torn and conflict prone settings obviously live in a situation where crisis-focused palliative care delivery is far from being an agenda. This unquestionably multiplies the victims' physical pains, social instabilities and metal crises. On the hand, the country has a huge social capital, such as folk-music therapy and spiritual counseling, which can have important implications for modern palliative care provision (e.g. community-based and home-based cares), especially during natural and manmade disasters. Yet, it requires systemic thinking and commendable holistic approaches to gauge the social capital into palliative care schemes, which are currently lacking in the country.

In fact, the Ethiopian health system has recognized the role of palliative care in pain management and relief. For instance, the Federal Ministry of Health (FMOH) has valued its importance, as described in hospital transformation guideline, and made it part of the trainings given to health professionals. Besides, the FMOH initiated the commencement of a project named ‘Pain Free Hospital Initiative (PFHI)’ that aims to provide teaching on pain assessment and control to physicians, nurses and pharmacists in hospitals located in Addis Ababa (7). A study also reported that palliative care was integrated into the healthcare services of two health institutions located in Addis Ababa (8).

Other palliative care providing schemes in Ethiopia include Hospice Ethiopia and Strong Hearts (8). The establishment of a palliative care unit at Jimma University Medical Center is another positive development. Palliative care trainings are also being available for prospective and serving health professionals. A palliative care technical groups assigned at the FMOH, mandated to coordinate the major palliative care delivery endeavors in the country. All these, nevertheless, are only promising starts, which fall short of meeting the immense palliative care needs of the Ethiopian nation.

As discussed before, the high prevalence of life-threating diseases such as cancer and HIV/AIDS (9,10), coupled with the worsening humanitarian crises, has made the number of palliative care seekers so overwhelming that the existing schemes cannot respond to it to a meaningful degree, leave alone fully. For example, the institutionalization of palliative care service in two out of 87 hospitals in Addis Ababa was found to be quite inadequate (8). It can also be questionable whether or not the available palliative care delivering schemes are able to respond to the diverse needs of those who have access to them.

Overall, the diverse and acute palliative needs of the Ethiopian populations remain hugely unmet in the context of worsening disease conditions and exponentially increasing number of victims of humanitarian crises such as conflict-inflicted calamities. Therefore, it is necessary to make extraordinary efforts, within all the daunting challenges, to create robust, cost-effective and context-specific palliative care services. This requires holistic approaches, collaborative efforts and wise utilization of all available resources, including the social capital.

EJHS is running successfully in this exceedingly demanding national situation, and this is the sixth regular issue of the year 2023. The issue contains…
